# Editorial: Expansion of the Genetic Code: Unnatural Amino Acids and their Applications

**DOI:** 10.3389/fchem.2022.958433

**Published:** 2022-08-08

**Authors:** Subhendu Sekhar Bag, Ishu Saraogi, Jiantao Guo

**Affiliations:** ^1^ Department of Chemistry, Indian Institute of Technology Guwahati, Guwahati, India; ^2^ Department of Chemistry, Indian Institute of Science Education and Research, Bhopal, India; ^3^ Department of Chemistry, University of Nebraska-Lincoln, Lincoln, NE, United States

**Keywords:** expansion of genetic code, unnatural amino acids (uAAs), tRNA synthetase library, amber codon, peptidomimetic, post-translational modification, codon reassignment, chemical protein synthesis

Every living organism encodes proteins using the same 20 natural amino acid building blocks, possessing a limited number of functional groups, in response to selective triplet codons. However, evidence of a large number of post-translational modifications in the protein cofactors and the rare occurrence of two unnatural amino acids, namely selenocysteine and pyrrolysine, clearly indicates the need for additional functional groups. A milestone achieved by (Offord, 1987; Kaiser, 1989) in the development of synthetic and semi-synthetic methods for the incorporation of unnatural amino acids into desired peptide or protein sequences paved the way for Schultz et al*.*, for developing a strategy towards the site-specific incorporation of unnatural amino acids into proteins, thereby expanding the genetic code. In 1989, *Schultz and coworkers* reported the introduction of an amber stop codon at the desired site, in a gene of interest, via site-directed mutagenesis (Noren et al., 1989). Translation of the corresponding mRNA was possible by means of amber-stop codon suppression (Guo et al., 2009). Since then, an increase in interest in pursuing research on expanding the genetic code has prompted scientists globally to design unnatural amino acids. Although many unnatural amino acids were found to be unsuitable for imparting novel functionalities in desired proteins, the expansion of genetic code has dramatically increased the functional potential of a wide variety of proteins and peptides alike. Thus, the translation of an expanded genetic code has the potential to produce semi-synthetic organisms with increased biodiversity and functionalities.

This special issue mainly focuses on the recent achievements at the junction of organic chemistry and molecular biology, especially towards expanding the genetic code. Here we highlight the synthesis of several unnatural amino acids, the generation of non-canonical tRNA synthetase libraries and tRNAs, site-specific incorporation of uAA into target proteins and the study of synthetic protein functions.

Expansion of the genetic code is known to increase or modify the functionality of proteins. Towards this end, Patrick O’Donoghue et al. reported the production of a site-specifically acylated variant of *Thioredoxin Reductase 1* (*TrxR1*), containing selenocysteine as the unnatural amino acid. The authors showed that the modified *TrxR1* enzymatic protein could provide resistance against oxidative damage even under oxidising conditions. It was further demonstrated that the acetylation of *TrxR1* could enhance the redox activity facilitating *TrxR1* to resist oxidative damage even in the presence of very reactive oxygen species.

In an effort toward engineering the active site of (*R*)-*amine transaminase* enzyme, Hyngdon Yun and his group reported the site-specific incorporation of non-canonical amino acid, *p*-benzoyl phenylalanince (pBpA), following the strategy of expansion of the genetic code. Thus, the replacement of a single phenylalanine residue at the 88th position of the active site resulted in a 15 times enhancement in activity for 1-phenylpropan-1-amine and an 8 times enhancement for benzaldehyde. In another instance, F86A/F88pBpA, a 30% increment in thermostability of the enzyme at 55°C was reported without any change in enzymatic activity. Therefore, Hyngdon Yun and coworkers clearly showed the enhanced functionalities of enzymes having expanded genetic codes.

The application of expansion of the genetic code in the study of acetylation was demonstrated by Chenguang Fan and coworkers. The enzyme aconitase has been known to catalyse the reversible conversion of citrate and isocitrate. The model organism *E. coli* is known to possess two isoforms of the enzyme, namely AcnA and AcnB. The authors showed that the acetylation of AcnA K684 resulted in a decrease in enzymatic activity, while the acetylation of AcnB K567 led to an increase in activity. This report provided the opportunity of generating proteins of diverse and altered functionality depending on the site-specific incorporation of an expanded genetic code.

Scientists have been trying to improve the interaction between codons and anticodons at in-frame amber-stop codons in order to improve yields of proteins with an expanded genetic code (Mala and Saraogi, 2022). However, reports of site-specific incorporation of unnatural amino acids at the N-termini of proteins are rare. Towards this end, Tae Hyeon Yoo et al. developed an orthogonal translation initiation system for site-specific incorporation of unnatural amino acids at the N-termini of proteins. Thus, a *Methanococcus jannaschii* tRNA^Tyr^ was engineered into an initiator tRNA by introducing identity elements of *E. coli* initiator tRNA. Using this system, they were able to site-specifically incorporate an unnatural amino acid *O*-propargyl-l-tyrosine (OpgY) at the translation initiation position by means of amber codon suppression. Further results indicated that this system was inactive towards the incorporation of uAA in response to any other internal TAG codon. To avoid misincorporation of Gln amino acid, the study was carried out only in the presence of *O*-propargyl-l-tyrosine (OpgY) amino acid.

Over the years, scientists have mainly introduced unnatural amino acids in response to a suppressed amber-stop codon. However, the strategy of sense codon reassignment leads to the incorporation of a wide variety of non-canonical amino acids, which may find their use in the advancement of protein engineering and bioorganic chemistry. In this line, John D. Fisk and his group reported the reassignment of an arginine sense codon, AGG, for the incorporation of tyrosine amino acid. The most efficient *M. jannaschii* tyrosyl tRNA synthetase variants capable of incorporating tyrosine in place of arginine were later selected and transplanted onto another *M. jannaschii* aaRS, evolved for the incorporation of an unnatural amino acid *p*-azidophenylalanine. This study opened up a new gateway of incorporation of newer amino acids by reassigning sense codons for the generation of novel recombinant proteins.

Besides protein engineering, unnatural amino acids find potential applications in designing peptidomimetic therapeutics with enhanced pharmacology, diagnostics and supra-molecular self-assembly/organogel. Over the years, several types of designer amino acids, including fluorescent triazolyl amino acids and scaffold amino acids (Bag et al., 2015) have been developed to synthesise peptidomimetics. Aminopyrazonolyl amino acid scaffold having hydrogen-bonded supramolecular self-assembling properties has been exploited by Nagendra Sharma et al. to showcase organogellation. Thus they have shown that a type of aminopyrazolone amino acid, namely O-alkylated ampyrone containing hybrid peptides, formed organogels after sonication in ethyl acetate: hexane solvent (1:3) under set parameters. Such APA-peptides also showcased the potential formation of hydrogen bonding, and are promising candidates to function as peptidomimetics.

The detailed study of oxidative post-translational modifications of proteins is very important. Such modifications are often reported in disease pathology and can serve as potential tools for predicting the disease. Methods to selectively detect proteins with expanded genetic code, mimicking oxPTM (oxidative post-translational modification) scenarios, are very essential because such oxPTM proteins have been known to result in functional changes that can eventually contribute to disease pathology. The accumulation of oxPTM nitrotyrosine is reported in over 100 proteins associated with diseases. This prompted Ryan A. Mehl and his coworkers to develop a nanobody selective for 3-nitrotyrosine modified 14-3-3 signalling protein. The same nanobody was reportedly less specific for nitrotyrosine present in other proteins. Using this strategy, the authors demonstrated the selectivity of a single nanobody to oxidative post-translationally modified protein targets, even in highly proteinaceous solutions. Nanobodies have immense potential to serve as powerful tools to study the complex intracellular dynamics of oxPTMs and their adverse pathological impacts.

Genetically encoded designer amino acids with electrophilic moieties are often found as excellent candidates for studying protein-protein interactions through cross-linking. Towards this end, an alkyl bromide-based unnatural amino acid (BprY) has been utilised by Xin Shu et al. to investigate protein-protein interactions under both *in vitro* and *in vivo* conditions. The BprY was found to target not only cysteine but also a broad range of nucleophilic amino acids to form protein-protein cross-links. The results were supported by the broad reactivity of BprY with the Affibody/Z protein complex. The same unnatural amino acid was also used to study the interaction of SUMO2 (Small Ubiquitin-like Modifier 2) and RNF111, which was devoid of any cysteine residues at the binding site. A total of 264 SUMO2 interacting proteins were captured and identified at a whole proteome level using the same uAA. Thus, the authors demonstrated that BprY and relevant alkyl halide unnatural amino acids could serve as excellent candidates to map protein-protein interactions. Additionally, Xuyu Liu and his group reviewed the use of synthetic thiol, and selenol-derived unnatural amino acids for the expansion of chemical protein synthesis. This review is an excellent resource for researchers seeking insights into the chemical synthesis of various analogues of thiolated and selenylated amino acid molecules. The expanded scope of native chemical ligations to assess homogeneously modified proteins in a highly efficient manner has also been highlighted. They have also shown the selected amino acids in the applications of the chemically synthesised thiolated and selenylated amino acids for the chemical synthesis of selected post-translationally modified peptides and proteins. Several strategies, such as native chemical ligation, have been explored for the synthesis of post-translationally modified proteins. The native chemical ligation strategy offers an exciting opportunity to construct libraries of protein therapeutics.

Overall, this thematic issue highlights the potential impact of unnatural amino acids in bioorganic chemistry and chemical biology. Reports on exploring unnatural amino acids toward the expansion of genetic code, generation of proteins with structural and functional diversity, and modulation of enzymatic activities have been demonstrated. Genetically encoded unnatural amino acids have also been showcased in designing peptidomimetic therapeutics and the study of protein-protein interactions. This special issue, thus, would have a significant impact on the advancement of research toward the expansion of the genetic code leading to the generation of proteins/enzymes with enhanced and diverse functionalities.
